# Positive correlation between genetic diversity and fitness in a large, well-connected metapopulation

**DOI:** 10.1186/1741-7007-6-46

**Published:** 2008-11-05

**Authors:** Sofie Vandewoestijne, Nicolas Schtickzelle, Michel Baguette

**Affiliations:** 1 Biodiversity Research Centre, Université catholique de Louvain, Place Croix du Sud 5, 1348 Louvain-la-Neuve, Belgium; 2 Muséum National d'Histoire Naturelle, Département Ecologie et Gestion de la Biodiversité, CNRS UMR 7179, 4 Avenue du Petit-Château, 91800 Brunoy, France

## Abstract

**Background:**

Theory predicts that lower dispersal, and associated gene flow, leads to decreased genetic diversity in small isolated populations, which generates adverse consequences for fitness, and subsequently for demography. Here we report for the first time this effect in a well-connected natural butterfly metapopulation with high population densities at the edge of its distribution range.

**Results:**

We demonstrate that: (1) lower genetic diversity was coupled to a sharp decrease in adult lifetime expectancy, a key component of individual fitness; (2) genetic diversity was positively correlated to the number of dispersing individuals (indicative of landscape functional connectivity) and adult population size; (3) parameters inferred from capture-recapture procedures (population size and dispersal events between patches) correlated much better with genetic diversity than estimates usually used as surrogates for population size (patch area and descriptors of habitat quality) and dispersal (structural connectivity index).

**Conclusion:**

Our results suggest that dispersal is a very important factor maintaining genetic diversity. Even at a very local spatial scale in a metapopulation consisting of large high-density populations interconnected by considerable dispersal rates, genetic diversity can be decreased and directly affect the fitness of individuals. From a biodiversity conservation perspective, this study clearly shows the benefits of both in-depth demographic and genetic analyses. Accordingly, to ensure the long-term survival of populations, conservation actions should not be blindly based on patch area and structural isolation. This result may be especially pertinent for species at their range margins, particularly in this era of rapid environmental change.

## Background

Habitat destruction and fragmentation result in decreased habitat patch size and increased habitat patch isolation. Consequences of this common and now well-studied phenomenon include, amongst others, modified community composition and structure, smaller population sizes and decreased population connectivity [1]. Both population size and connectivity significantly affect population genetic diversity, which can subsequently affect fitness. Indeed, as population size decreases, genetic stochasticity increases, resulting in increased allele fixation with each generation due to higher genetic drift. Homozygosity also increases due to a higher frequency of mating among relatives. Decreased connectivity augments the effects of small population sizes because of a reduction in successful dispersal events. Consequently, no or few alleles arrive to enhance the existing gene pool. Accordingly, smaller and more isolated populations are generally characterized by low genetic diversity (e.g. [2]). Theory predicts that loss of genetic diversity generates adverse consequences for fitness, and subsequently for population demography, in small isolated populations [3], which is indeed confirmed by empirical evidence [2,4-6]. Hence, the major goal of conservation managers is to maintain minimum viable population sizes not only by increasing population size but also by enhancing connectivity between populations (e.g. [7]).

**Figure 1:**
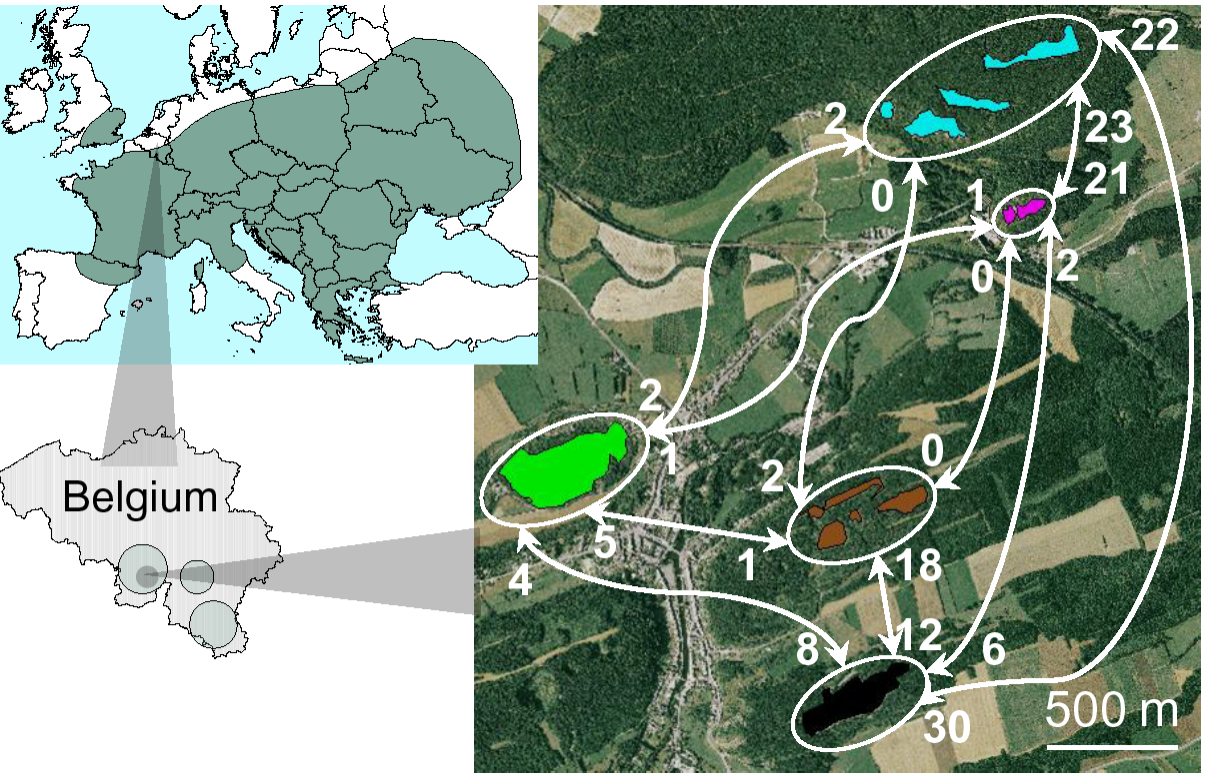
***Polyommatus coridon *****worldwide (a) and Belgian (b) distribution range.** Study sites (c) with number of observed between-population movements (males and females combined) indicated in white.

In this study, we analysed the relationship between genetic diversity and fitness in a butterfly metapopulation within a highly fragmented landscape. Contrary to most studies relating genetic diversity to fitness, and despite the high amount of habitat loss and fragmentation within the study area, the chosen model species still has local populations of relatively large sizes and high connectivity. Both demographic and genetic techniques were used to estimate genetic diversity and fitness in five populations of the chalk-hill blue butterfly *Polyommatus coridon *(Poda) (Lycaenidae, Lepidoptera) situated in southern Belgium, at the northern edge of the species distribution range (Figure 1). These five populations were the largest populations in the area. Several other suitable sites were visited in the neighbourhood, but no or very few *P. coridon *individuals were observed. *Polyommatus coridon *is a thermophilic univoltine habitat specialist of calcareous grasslands [8,9], where caterpillars feed exclusively (T. Schmitt, personal communication) on the horseshoe vetch, *Hippocrepis comosa*. It is one of the six most characteristic butterfly species of European calcareous grasslands [10]. Within the Walloon region (i.e. southern Belgium), *P. coridon *is classified as rare and in decline, and its distribution range has regressed by 70% since the 1950s [11]. Consequently, it is a species of high conservation priority.

Calcareous grasslands are biodiversity hotspots not only because they are amongst the richest plant and insect communities in Europe [12], but also because they harbour numerous species from diverse regions, including Southern Mediterranean and Eastern Asia. They are also a central issue in nature conservation management in Europe because most of the calcareous grasslands are semi-natural habitats, i.e. created by human activity. Since the beginning of the 20th century, calcareous grasslands and their associated communities have declined severely following the progressive abandonment of agropastoral practices due to a lack of economical interest, land use intensification, and urbanisation [13]. Indeed, most former calcareous grasslands have been afforested or have developed into woodlands by natural succession [14]. Our study area reflects this general trend [15]. In 2004 only 4.76% of the calcareous grassland surface of 1905 remained. This drastic decrease in calcareous grassland area is accompanied by a sharp decrease in average patch area (from 7.8 ha in 1905 to 0.8 ha in 2004), in patch number (22 large patches in 1905, 45 small patches in 1965, and 10 small patches in 2004), and in average patch connectivity (connectivity in 2004 is only 6% of that in 1905). Habitat loss and fragmentation in this region has had a profound effect on the butterfly community, including the extinction of specialist species and a subsequent increase in the over-representation of generalist species. Butterfly abundance, for both specialist and generalist species (based on common versus rare status), has also decreased significantly during this time period within the study region [15]. These modifications have also been observed elsewhere (i.e. on western German calcareous grasslands [16]). Consequently, calcareous grasslands are of high conservation priority, not only because they are biodiversity hotspots but also because of their historical (and cultural) heritage. The fact that the study system is found at the edge of *P. coridon*'s distribution range does not make it less important for conservation management, especially when the main factors driving extinction are external (e.g. [17]).

The primary aim of this study was to analyse the relationship between genetic diversity and fitness in a species of high conservation value within a landscape that has deteriorated significantly over the last century. Demographic data were used to estimate population size, effective population connectivity (number of successful dispersal events) and a fitness component, lifetime expectancy. We then investigated how genetic diversity affected lifetime expectancy. Lifetime expectancy is an important fitness component in insects because it directly affects lifetime reproductive success of many species [18], particularly polygamous species mating throughout their life, as *P. coridon*. Indeed, a longer life span will allow a greater number of matings (both sexes of *P. coridon *mate more than once). Furthermore, *P. coridon *females with a higher lifetime expectancy will lay more eggs since they lay eggs singly and continually until they die, but need to attain a certain maturity after hatching before being able to lay eggs, and lay fewer eggs in early life compared with later on [9].

Additionally, since this study was conducted within the framework of a nature conservation project with concrete management guidelines as one of the main objectives, we relate our demographic and genetic results to surrogate indices often used in applied conservation studies: habitat patch area and habitat quality descriptors as surrogates for population size, and structural connectivity as a surrogate for the intensity of dispersal and associated gene flow. Central to the metapopulation theory, the basic idea is that increased patch size and/or connectivity lead to increased population sizes (e.g. [19]). Populations of larger size are expected to be more viable because they are subjected less to demographic, genetic, and environmental stochasticity [20]. The long-term viability of populations is also expected to decrease with increased geographic isolation. Indeed, due to a decrease in successful dispersal events, locally isolated populations will not benefit from population replenishment by immigrants, which is necessary for population subsistence when the birth rate is exceptionally low or the death rate high [21]. The supply and exchange of genes (alleles) also decreases in isolated populations, resulting in lower genetic diversity and increased inter-population differentiation [22] (but see [23]).

The causal link between genetic diversity and fitness cannot be demonstrated without large experimental breeding and/or translocation manipulations at the population level, which is almost impossible to put into practice, especially with a threatened species. In such study systems it is therefore often necessary to correlate the variation observed in several traits, and we used this approach in our study; this correlation approach often generates rich conclusions (e.g. [24]). Obtaining high quality estimates of genetic, demographic, and dispersal data was a clear prerequisite to be able to assess existing relations between these three kinds of trait. This required a huge investment in data collection and analysis, forcing a trade-off with the number of populations analysed in this study. The relatively small number of populations (five) studied here placed certain limits on the power of statistical tests. As a consequence, we did not interpret the very statistical level associated with the test of any single particular correlation coefficient, but focused on patterns emerging from sets of correlations involving alternative measures of genetic diversity.

Demography of the five populations was studied with the Capture-Mark-Recapture (CMR) methodology. The large CMR data set (7228 (re)captures of 2789 different individuals) was analysed using high-performance statistical procedures to obtain estimates of demographic parameters (survival and birth rates). Lifetime expectancy at birth (*LTE*), i.e. the number of days a newborn butterfly is expected to live, was computed for each of the five populations from these values of survival and birth rates. Genetic diversity was evaluated using three alternative and partially redundant measures based on 30 ISSR (intersimple sequence repeat) loci: *He *(expected heterozygosity, calculated from the null allele frequency and based on the Hardy-Weinberg equilibrium, with allele frequencies calculated from null homozygote frequencies assuming panmixia and corrected for dominance), *Div *(average gene diversity, i.e. mean number of pairwise haplotype differences), and *PPL *(percentage polymorphic loci). From here on, we refer to *He*, *PPL *and *Div *collectively when using the term 'genetic diversity'.

## Results

Population sizes (total over the flight period) varied between 2301 and 171 individuals (sexes pooled), and lifetime expectancy *LTE *varied from 8.46 to 10.86 days (Table 1). More detailed demographic results are out of the scope of the present paper; they are therefore not displayed here.

**Table 1:** Genetic, demographic and ecological data collected for five populations of *Polyommatus coridon *in the Walloon region (i.e. southern Belgium)

		Montagne au Buis	Roche à Lomme	Abannets	Tienne Breumont	Fondy des Chiens	Entire data set
Genetic data	Sample size (individuals)	28	31	25	30	30	144
	*He*	0.333 ± 0.056	0.309 ± 0.066	0.302 ± 0.070	0.294 ± 0.063	0.313 ± 0.060	0.321 ± 0.056
	*Div*	0.364 ± 0.084	0.328 ± 0.072	0.307 ± 0.078	0.339 ± 0.076	0.345 ± 0.077	0.347 ± 0.033
	*PPL*	96. 7	90	90	86.7	96.7	96.7
							
Demographic data	Census population size	1620 ± 84	384 ± 106	171 ± 24	630 ± 130	2301 ± 130	
	*LTE *(days)	10.86 (9.98–12.03)	9.43 (7.71–12.06)	8.86 (7.02–11.60)	8.46 (7.41–9.71)	9.50 (8.65–10.49)	
	Number of immigrants	47	24	21	12	56	
	Number of emigrants	55	30	17	12	46	
							
Landscape and environmental data	Area	4.3	0.67	2.77	8.03	3.97	
	Structural connectivity	0.0035	0.0155	0.0146	0.0038	0.0087	
	Microclimatic conditions	-0.228 ± 0.614	-1.624 ± 1.501	1.449 ± 0.595	0.094 ± 0.565	-0.191 ± 0.739	
	Nectar abundance	39.350 ± 7.417	38.583 ± 12.795	35.944 ± 8.048	26.000 ± 3.406	41.391 ± 7.349	
	Host plant abundance	5.750 ± 3.979	5.750 ± 7.112	3.810 ± 5.476	0.059 ± 0.120	1.826 ± 3.603	

*He*: expected heterozygosity, *Div*: average gene diversity, *PPL*: percentage of polymorphic loci, *LTE*: lifetime expectancy. A 95% confidence interval is given when available (± half length if symmetrical, lower and upper values when asymmetrical).

The average gene diversity *Div *over all ISSR loci and for all populations was 0.347 ± 0.176 (Table 1), indicating that about 35% of the loci differed between all pairs of ISSR genotypes. The expected heterozygosity *He *was 0.321 and the percentage of polymorphic loci *PPL *was 96.7%. As expected, the three measures of genetic diversity were all positively correlated to each other, although strictly speaking only the *He-PPL *correlation was statistically significant at the 0.05 level (*He*-*Div*: *r *= 0.700, *p *= 0.188; *He*-*PPL*: *r *= 0.949, *p *= 0.014; *Div*-*PPL*: *r *= 0.632, *p *= 0.252). Population differentiation was low but significant: 0.0268 and 0.0225 (*p *= 0.001) for *F*_*st *_and *θ*_*st*_, respectively. Fondry des Chiens and Abannets were the most genetically differentiated populations (Table 2). No significant (geographic) isolation by distance was detected (*r *= -0.040, *p *= 0.495).

**Table 2:** Population pairwise *θ*_*st *_values. Bold values signify a significant *θ*_*st *_value at the 0.05 level

	Montagne au Buis		Roche à Lomme		Abannets		Tienne Breumont	
	*θ*_*st*_	*p*	*θ*_*st*_	*p*	*θ*_*st*_	*p*	*θ*_*st*_	*p*
Roche à Lomme	0.013	0.132						
Abannets	**0.027**	**0.039**	0.013	0.149				
Tienne Breumont	0.007	0.237	0.010	0.165	**0.025**	**0.043**		
Fondry des Chiens	**0.038**	**0.005**	**0.056**	**0.001**	**0.037**	**0.010**	**0.050**	**0.001**

Patterns of correlations were clearly visible among all the genetic, demographic, and dispersal measures: whereas correlations presented in Table 3 (genetic diversity, *LTE*, population size and dispersal rate) are high (10/11 are higher or equal to 0.63), those presented in Table 4 and Table 5 (genetic diversity and *LTE *with surrogates for population size and dispersal rate) are substantially lower (only 1/20 is higher than 0.63).

**Table 3:** Correlation between genetic diversity, lifetime expectancy, population size and dispersal rate

	Lifetime expectancy (*LTE*)		Census population size (*N*)		Dispersal rate (number of immigrants)	
	*r*	*p*	*r*	*p*	*r*	*p*
Expected heterozygosity (*He*)	**1.000**	**<0.0001**	0.600	0.285	**1.000**	**<0.0001**
Gene diversity (*Div*)	0.700	0.188	**0.900**	**0.037**	0.700	0.188
Percentage of polymorphic loci (*PPL*)	**0.949**	**0.014**	0.632	0.252	**0.949**	**0.014**
Lifetime expectancy (*LTE*)	**-**	**-**	0.600	0.285	**1.000**	**<0.0001**

Spearman coefficients (*r*) in bold are statistically significant at the 0.05 level.

**Table 4:** Correlation between genetic diversity, lifetime expectancy, and surrogates of population size: patch area, microclimatic conditions, nectar and host plant abundance.

	Patch area		Microclimatic conditions		Nectar abundance		Host plant abundance	
	*r*	*p*	*r*	*p*	*r*	*p*	*r*	*p*
Expected heterozygosity (*He*)	-0.100	0.873	-0.600	0.285	0.300	0.624	0.616	0.269
Gene diversity (*Div*)	0.600	0.285	-0.400	0.505	-0.200	0.747	0.051	0.935
Percentage of polymorphic loci (*PPL*)	-0.105	0.866	-0.369	0.541	0.527	0.362	0.433	0.467
Lifetime expectancy (*LTE*)	-0.100	0.873	-0.600	0.285	0.300	0.624	0.616	0.269

Spearman coefficients (*r*) in bold are statistically significant at the 0.05 level.

**Table 5:** Correlation between genetic diversity, lifetime expectancy, and surrogate of dispersal rate: structural connectivity

	Structural connectivity	
	*r*	*p*
Expected heterozygosity (*He*)	-0.300	0.624
Gene diversity (*Div*)	-0.800	0.104
Percentage of polymorphic loci (*PPL*)	-0.264	0.668
Lifetime expectancy (*LTE*)	-0.300	0.624

Spearman coefficients (*r*) in bold are statistically significant at the 0.05 level.

**Figure 2:**
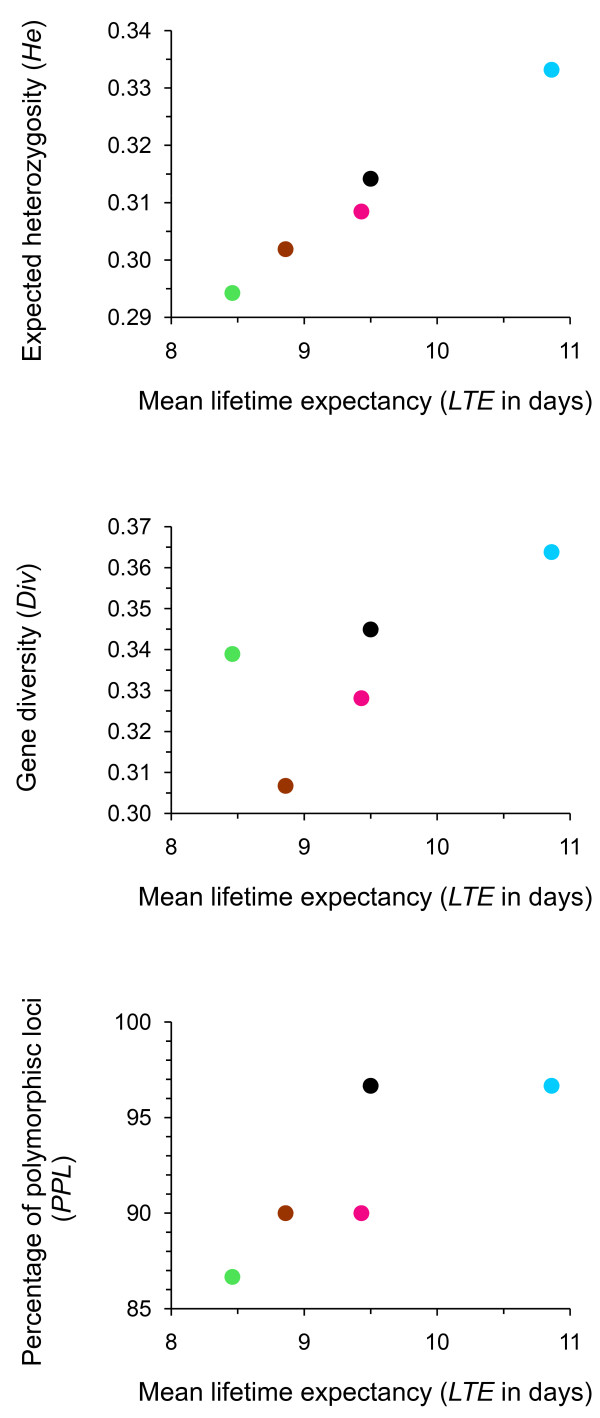
**Genetic diversity was positively correlated with lifetime expectancy (*LTE*)**. Butterflies from local populations with the highest genetic diversity had a *LTE *up to c. 25% higher, directly affecting their individual fitness through lifetime reproductive success. Colours refer to sites in Figure 1.

**Figure 3:**
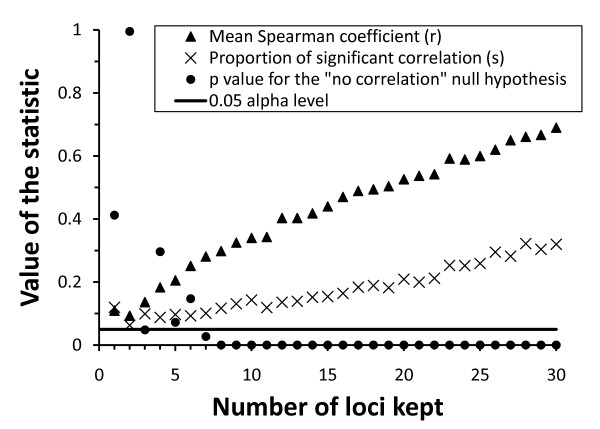
**The correlation between genetic diversity, represented by the expected heterozygosity *He*, and lifetime expectancy *LTE *was robust to the estimation errors existing in both estimates, even for a large decrease in the number of loci considered, indicating that despite correlations being based on five populations only, the statistical power was sufficient, given the existing estimation errors**. A bootstrap procedure with 1000 random drawings was used to obtain these results (see text for details).

Variations between populations in genetic diversity were positively correlated to variations in *LTE *(Table 3; Figure 2). This correlation was highly significant for *He*. While not significant at the 0.05 level, *Div *and *PPL *also showed largely positive covariation with *LTE*. The example of the *He*-*LTE *correlation is used to show that this correlation remained largely unaltered when the uncertainties in the estimates of *He *and *LTE *were fully taken into account, and is robust to a large decrease of the number of loci on which genetic diversity was computed (Figure 3). This positive relation implies that butterflies from populations with the highest genetic diversity had a mean lifetime expectancy up to c. 25% higher than butterflies from populations characterised by a low genetic diversity.

Genetic diversity was positively correlated with population size (Table 3), but not with its surrogates (Table 4). Likewise, genetic diversity was positively correlated with number of immigration dispersal events (Table 3) but not with its surrogate (Table 5). No correlation was observed between *LTE *and population size or its surrogates (Tables 3 and 4); *LTE *was positively correlated with number of immigration events, but not with its surrogate (Tables 3 and 5); *LTE *was also positively correlated with the number of emigration events (*r *= 0.900, *p *= 0.037), but not with the net number of immigrants (immigration minus emigration: *r *= 0.300, *p *= 0.624).

## Discussion

Using both demographic and genetic techniques on a metapopulation of *P. coridon *consisting of large and well connected populations, three conclusions were reached: (1) the most important conclusion is that fitness of butterflies positively correlated with genetic diversity; (2) immigration rate and population size were also positively correlated with genetic diversity; and (3) CMR-based estimates of population size and dispersal rates were better indicators of genetic diversity than were some of their more conventionally used surrogates: habitat patch area, various measures of habitat quality, and geographic patch isolation.

Lower genetic diversity was coupled to a sharp decrease in adult lifetime expectancy (*LTE*): *P. coridon *butterflies of some populations lived for up to 25% (2.4 days) longer on average than their counterparts of other populations, therefore significantly increasing their individual fitness. *LTE *is definitely a major component of individual fitness via lifetime reproductive success in this species. Indeed, both sexes mate more than once and females with a higher *LTE *will lay more eggs, because females lay eggs singly and continually until they die but need to attain a certain maturity after hatching before being able to lay eggs [9].

Despite the limits inherent to the correlational approach and the restricted number of populations sampled in this study, the validity of our conclusions is supported by three main elements. First, the accuracy of estimates for genetic, demographic and dispersal parameters was very high compared with the standard level achieved in other studies. Genetic diversity measures were based on a relatively large number of loci. Demographic and dispersal measures were based on a very large CMR dataset. *LTE *not only integrates survival over the entire adults' life-span (daily survival changed with time) but also incorporates the variation in birth rate over time (which followed a parabolic distribution), which results in one robust and comprehensive value of average adult lifetime expectancy per population. Second, the *He*-*LTE *correlation remained highly significant even when the errors associated with demographic and genetic estimates were taken into account, indicating that the restricted number of populations was sufficient for detection of trends, despite the estimation errors. And third, clear trends of similar and consistent correlations were observed with alternative measures of genetic diversity.

Furthermore, although artefacts could potentially have influenced the observed correlation between fitness and genetic diversity, they do not alter the validity of our conclusions. First, the issue of population structure (i.e. significant population differentiation) has been raised as one of the most likely causes of association between genetic diversity and fitness related traits. Indeed, sampling individuals from different geographic origins could confound associations because of environmental heterogeneity at the sites [25]. We are convinced that this explanation can be ruled out in the present case for several reasons. First of all, the studied populations were all located within a very restricted geographic area and were readily identifiable because of the very specific ecological requirements of *P. coridon*'s host plant. Additionally, CMR data confirmed that the five populations were in fact independent but interconnected units: dispersal within populations (intra-patch movements) was more than 10 times as large as between population dispersal, and none of the populations functioned as source or sink. Genetic analyses confirmed these results: population differentiation was significant but extremely low. Low population differentiation at local and regional scales is typical for this species [26]. Second, a positive correlation between genetic diversity and *LTE *could also be due to the recognition by *P. coridon *adults of differential habitat quality among patches. Indeed, prime habitat would facilitate long life spans in butterflies living there and attract, and keep, many individuals from outside the patch, leading to increased genetic diversity. As a consequence, populations in prime habitat will show an increased *LTE *and an increased genetic diversity, both caused by habitat quality, and the link between genetic diversity and *LTE *might not express an effect of genetic diversity on fitness. If this scenario were the case in our study, we would expect to find both a positive relation between *LTE *and some of the usual habitat quality descriptors (habitat area, microclimate, adult nectar resources, host plant abundance, structural connectivity), and between *LTE *and the net number of immigrants (immigrants minus emigrants), which was not the case. Some of the harshest conditions were even found on the site characterised by the highest genetic diversity and *LTE*. Indeed, vegetation in this site had completely dried up long before the end of the *P. coridon *flight season, including its host plant (SV, personal observation). Third, *LTE *might be biased by emigration rate, because CMR analyses consider as identical the death or the emigration of an individual, both being disappearance events that decrease the estimate of survival rate (and then *LTE*). In this case, a negative trend between *LTE *and the number of emigration events is expected. However, emigration rate from the five populations was relatively low compared with the amount of demographic data (3% of captures), and a positive correlation was observed between *LTE *and the number of emigration events, i.e. in the opposite direction to the expected bias. The bias of *LTE *due to emigration decreased differences between populations in *LTE *instead of exaggerating them, and the observed positive correlations between *LTE *and genetic diversity were therefore conservative.

So far, the interaction between genetic diversity and fitness (i.e. survival and/or reproductive success) has only been revealed when effective population size becomes low and/or populations are extremely isolated (e.g. [27,28]). Small population sizes favour inbreeding behaviour and allow genetic drift to erode genetic diversity, while increased isolation limits gene flow, i.e. arrival of new alleles and non-related individuals. Whether the positive genetic diversity-lifetime expectancy correlation is due to heterosis, where heterozygous genotypes are superior to any of the homozygous genotypes, or due to partial dominance, where decreased allelic diversity leads to the expression of recessive or partially recessive deleterious alleles, cannot be determined with the present results but certainly deserves further research.

In the present study, demographic analyses indicated that the populations were neither small nor isolated. Although estimating effective population size (*Ne*) remains difficult, detecting inbreeding effects in population sizes of several hundreds to several thousands of individuals may suggest that *Ne*s were only a tiny fraction of census population sizes (*N*) in this study area. The same pattern of decrease in genetic diversity in small and isolated local populations was recently detected within a metapopulation of lesser kestrel (*Falco naumanni*) (Fleischer) (Aves, Falconidae), a vagile and migratory raptor with high dispersal potential [29]. Altogether, these pieces of evidence support the idea that there is a considerable difference between the total and the effective population size in metapopulations.

The second conclusion of our study is that number of dispersal events positively correlated with genetic diversity. That increased functional connectivity leads to greater genetic diversity is expected and was also detected in a large scale study of *P. coridon *[30]. However, it is quite surprising to detect this relationship at such a small spatial scale and with a relatively high connectivity between populations. Likewise, genetic diversity was positively correlated to population size. Larger population sizes are indeed generally more resistant to genetic erosion.

The third conclusion concerns the widespread use of surrogate variables. Building on the metapopulation theory, many conservation studies use patch area and structural connectivity measures as indicators of population size and isolation, respectively, two components with major demographic and genetic effects on the short- and long-term viability of species systems (e.g. [31-33]). Our study hints that these measures may not always be very reliable surrogates (see also [34] for patch area). Patch area and a series of well chosen habitat quality descriptors failed to reveal the links observed when a high quality CMR-based estimate of population size was used; similarly, structural connectivity failed to reveal the link observed when numbers of immigrants were used. This latter point connects to the fact that structural connectivity estimates do not capture the intra-specific variation in individual behaviour related to mobility, which has been shown to be strongly affected by both landscape fragmentation and metapopulation history (e.g. [35-38]). These results therefore suggest that demographic (such as CMR) studies provide more realistic and reliable indicators necessary for conservation programmes to be effective in the long-term. Unfortunately, they also necessitate much more investment.

## Conclusion

A positive correlation between genetic diversity and mean lifetime expectancy was detected at a very local scale in a metapopulation consisting of large high-density populations interconnected by considerable dispersal rates. Even in such a system, genetic diversity can be decreased and directly affect the fitness of individuals. From a biodiversity conservation perspective, this study clearly demonstrates the benefits of both in-depth demographic and genetic analyses and highlights that in order to ensure the long-term survival of the populations conservation actions should not be solely based on patch area and structural isolation. This result may be especially pertinent for species at their range margins, particularly in this era of rapid environmental change.

## Methods

### Demography

During the entire 2003 flight period (end of June to beginning of September), the demography of five *P. coridon *populations was studied with the CMR methodology. The five study sites, delimited on the basis of favourable habitat and topographic features, were visited as often as possible (weather permitting). Every encountered imago was captured with a net, individually marked with a permanent pen and immediately released. For each (re)capture, the following data were recorded: tag number, sex, age (estimated through wing wear), date and hour, site and patch.

Estimates of demographic parameters (daily survival rate *φ*_*t*_, capture rate *p*_*t*_, recruitment rate *b*_*t*_, daily population size *N*_*t*_, and total population size *N*) were obtained for the five populations separately, by analyzing the large CMR data set (7228 captures of 2789 individuals) using the constrained linear models method, with AIC-based [39] model selection procedure: Cormack-Jolly-Seber type model selection for survival and catchability and Jolly-Seber type model selection for survival, catchability and recruitment with MARK software [40]. This procedure (described in detail with references in [41]) is currently one of the best techniques to obtain estimates of both survival and birth rates in an open population, and is based on the selection of the best model among a set of candidate regression models describing variations (in the present case temporal) in demographic parameters. Sexes were pooled to enable comparisons with the genetic results.

Daily lifetime expectancy (*LTE*_*t*_), i.e. the number of days a newborn butterfly is expected to live, was computed from a virtual life table constructed from estimates of daily survival rate (*φ*_*t*_) using the formula:

$$LTE_{t}=\sum_{t=1}^{k}S_{t}/S_{t}$$

with *S*_*t *_= *S*_*t*-1_·*φ*_*t*-1 _and *S*_1 _being an arbitrarily fixed initial population size (not affecting the resulting estimate of *LTE*_*t*_). For each butterfly population, the mean *LTE *at birth was computed by weighting daily values of *LTE*_*t *_by daily number of births (*B*_*t*_, with *B*_*t *_= *b*_*t*_·*N*). Its 95% confidence interval was computed from the distribution of 1000 random drawings of *φ*_*t *_and *B*_*t *_from their variance-covariance matrices.

Numbers of dispersing individuals (immigrants and emigrants) were calculated directly from the CMR data set. The number of immigrants was used to quantify the dispersal rate as it reflects the potential amount of gene flow between populations.

### Genetics

Samples (between 25 and 31 per population) were collected, during the 2003 field season, using a non-invasive sampling technique: a tiny fraction of the wing or one leg per butterfly was sampled and analysed using dominant DNA neutral markers, i.e. intersimple sequence repeat [42]. Reactions were standardised and care was taken to create identical experimental conditions for all samples (PCR reactions were run on the same thermal cycler, identical products and concentrations were used for each run, etc.) Negative controls were used continually to check for contamination and amplifying the same samples on different days tested reproducibility. Product concentrations and PCR reaction parameters were similar to those used in [43]. PCR products were separated on 1.6% agarose gels (TBE buffer) that were run for 225 minutes at 100 V. Several DNA size standards were run on every gel to aid identification of the target bands. Ethidium bromide staining was used to visualise band patterns using GelDoc (Bio-Rad). Three ISSR primers out of 21 were selected based on the polymorphism and reproducibility of the bands that they generated: 809, (AG)_8_G; 816, (CA)_8_T; 848, (CA)_8_RG. Only primers with 100% reproducible bands were considered. Primers 809, 816, and 848 produced, respectively, 9, 11, and 14 bands that could be unambiguously scored, and were reproducible and polymorphic. Of the 34 polymorphic loci, four were dropped from analyses due to linkage disequilibrium. Results of analyses based on biased and unbiased allele frequencies [44] were very similar; therefore, we report only results based on the latter. Every sample was characterised by a different ISSR genotype.

Genetic diversity was quantified for each population by three measures: (1) expected heterozygosity *He*, (2) average gene diversity over all loci *Div*, and (3) percentage of polymorphic loci *PPL*. Allele frequencies were calculated from null homozygote frequencies assuming panmixia and corrected for dominance [44] using TFPGA 1.3 (Tools for Population Genetic Analyses [TFPGA] 1.3: A Windows Program for the Analysis of Allozyme and Molecular Population Genetic Datat, by MP Miller, 1997). Using these allele frequencies, *He *was calculated from the null allele frequency and based on the Hardy-Weinberg equilibrium. *PPL *(99% criterion) and genetic structuring (*θ*_*st*_) were calculated using the same program. We also used genotype data directly to obtain *Div*, the mean number of differences between all pairs of genotypes divided by the number of loci, and to derive *F*_*st *_from the variance components (AMOVA) using ARLEQUIN [45]. *He *and *Div *are independent estimates of genetic diversity since the former is based on allele frequencies and the latter on haplotype identity. *He *and *PPL *are probably correlated by nature because both depend on the presence or absence of polymorphism at the locus level. A Mantel test was used to assess the association between Nei's unbiased (1978) genetic matrix and the geographical distance matrix.

### Habitat network descriptors

The following habitat patch descriptors were measured: area, connectivity, a summary variable describing microclimatic conditions, abundance of nectar sources, and host plant abundance.

Patch area and inter-patch distances were calculated using aerial photographs (year 2000) with ArcGIS 9 software . Structural connectivity of a habitat patch was quantified according to its relation to all other patches in the study system:

$$S_{j}=\sum_{k\neq j}e^{-d_{jk}}A_{k}$$

where *d*_*jk *_is the distance between patch *j *and patch *k*, and *A*_*k *_the area of patch *k *[46]. Plant species abundance and diversity were inventoried for a total of 125 one metre squared plots within the *P. coridon *study system [47]. Microclimatic conditions were summarised by the first axis (PRIN1) of a principal component analysis (PCA) performed on eight descriptors, including four descriptors inferred by pooling knowledge on the vegetation composition with Ellenberg values of individual plant species (light L, temperature T, humidity F, and nutrient acidity N: [48]). This first component explained 61.55% of the variation existing in the original data. An increase along this axis represented an increase in bare ground, bare rocks, light and temperature, and a decrease in humidity, nutrient acidity, soil depth and bryophyte abundance. A higher value therefore represented a more xeric microclimate. A plant species was classified as a potential adult food resource by combining information concerning its nectar production and its flowering period, and field observations. Host plant abundance was based on measured *H. comosa *abundance.

### Statistical analyses

We quantified the correlation between the three estimates of genetic diversity (*He*, *Div*, and *PPL*) and the mean lifetime expectancy *LTE*, and the correlation of these four variables with (1) population size *N *and dispersal rate (number of immigrants and emigrants), (2) surrogates of population size (patch area, microclimatic conditions, nectar and host plant abundance), and (3) surrogate of dispersal rate (structural connectivity). As there was no a priori reason to believe that these would be linear correlations, rank (Spearman), instead of linear (Pearson), correlation coefficients were used throughout this study. No correction procedure for multiple testing has been used because we did not base our conclusions on a sharp interpretation of the significance level of each correlation, but on the general trend emerging from redundant correlations, as already mentioned.

However, we designed a bootstrap procedure [49] to assess how the most important relation between these variables, the one between genetic diversity (represented here by *He*) and *LTE*, was sensitive to the error present in the estimates of these variables. This procedure had a double aim: (1) to take into account the unequal precision of each population-specific estimate of average *He *and *LTE*, and (2) to test the robustness of the genetic data. Concerning *He*, a subset of the 30 loci was randomly selected and the mean computed for each population; subsets of 30 loci to only one locus were considered. On the other hand, *LTE *was computed for each population from randomly generated values of demographic parameters *φ*_*t *_and *B*_*t *_using their respective variance-covariance matrices. The Spearman's correlation coefficient *r *between population *He*-*LTE *means was then computed. This procedure was repeated 1000 times to construct the distribution of *r*. The mean *r *was reported as covariation measure between the two parameters studied. Statistical significance of this correlation (showing that it significantly differed from zero), however, was based on the probability of obtaining the observed proportion *s *(in the 1000 simulations) of significant correlations by chance. The distribution of *s *under the null hypothesis of no correlation was also obtained by resampling, with observed values for the site populations randomly shuffled, breaking any existing correlation. This shuffle procedure was repeated another 1000 times for each of the 1000 simulations and the *p*-value for the test was computed as the proportion of random associations with a proportion *s *of significant correlations greater than or equal to the observed *s*.

## Authors' contributions

SV and MB designed the research; SV performed the research; SV and NS analysed the data; SV, NS, and MB wrote the paper. All authors contributed equally to this work.

## References

[B1] Fahrig L (2003). Effects of habitat fragmentation on biodiversity. Ann Rev Ecol Evol Syst.

[B2] Frankham R (2005). Genetics and extinction. Biol Conserv.

[B3] Lande R (1988). Genetics and demography in biological conservation. Science.

[B4] Madsen T, Stille B, Shine R (1996). Inbreeding depression in an isolated population of adders Vipera berus. Biol Conserv.

[B5] Saccheri I, Kuussaari M, Kankare M, Vikman P, Fortelius W, Hanski I (1998). Inbreeding and extinction in a butterfly metapopulation. Nature.

[B6] O'Grady JJ, Brook BW, Reed DH, Ballou JD, Tonkyn DW, Frankham R (2006). Realistic levels of inbreeding depression strongly affect extinction risk in wild populations. Biol Conserv.

[B7] Crooks KR, Sanjayan M (2006). Connectivity Conservation.

[B8] Asher J, Warren MS, Fox R, Harding P, Jeffcoate G, Jeffcoate S (2001). The Millennium Atlas of Butterflies in Britain and Ireland.

[B9] Bink FA (1992). Ecologische Atlas van de Dagvlinders van Noordwest-Europa.

[B10] Van Swaay CAM (2002). The importance of calcareous grasslands for butterflies in Europe. Biol Conserv.

[B11] Fichefet V, Barbier Y, Baugnée J-Y, Dufrêne M, Goffart P, Maes D, Van Dyck H (2008). Papillons de jour de Wallonie (1985–2007).

[B12] WallisDeVries MF, Poschlod P, Willems JH (2002). Challenges for the conservation of calcareous grasslands in northwestern Europe: integrating the requirements of flora and fauna. Biol Conserv.

[B13] Balmer O, Erhardt A (2000). Consequences of succession on extensively grazed grasslands for central European butterfly communities: Rethinking conservation practices. Conserv Biol.

[B14] Poschlod P, WallisDeVries MF (2002). The historical and socioeconomic perspective of calcareous grasslands – lessons from the distant and recent past. Biol Conserv.

[B15] Polus E, Vandewoestijne S, Choutt J, Baguette M (2007). Tracking the effects of one century of habitat loss and fragmentation on calcareous grassland butterfly communities. Biodivers Conserv.

[B16] Wenzel M, Schmitt T, Weitzel M, Seitz A (2006). The severe decline of butterflies on western German calcareous grasslands during the last 30 years: A conservation problem. Biol Conserv.

[B17] Thomas CD, Bulman CR, Wilson RJ (2008). Where within a geographic range do species survive best? A matter of scale. Insect Conserv Diversity.

[B18] Thornhill R, Alcock J (1983). The Evolution of Insect Mating Systems.

[B19] Hanski I (1999). Metapopulation Ecology.

[B20] Shaffer ML (1981). Minimum population sizes for species conservation. BioScience.

[B21] Hanski I, Gilpin ME (1991). Metapopulation dynamics: brief history and conceptual domain. Biol J Linn Soc.

[B22] Frankham R (1998). Inbreeding and extinction: island populations. Conserv Biol.

[B23] Nève G, Barascud B, Descimon H, Baguette M (2008). Gene flow rise with habitat fragmentation in the bog fritillary butterfly (Lepidoptera: Nymphalidae). BMC Evol Biol.

[B24] Fjerdingstad EJ, Schtickzelle N, Manhes P, Gutierrez A, Clobert J (2007). Evolution of dispersal and life history strategies – *Tetrahymena *ciliates. BMC Evol Biol.

[B25] Slate J, Pemberton J (2006). Does reduced heterozygosity depress sperm quality in wild rabbits (*Oryctolagus cuniculus*)?. Curr Biol.

[B26] Schmitt T, Seitz A (2002). Influence of habitat fragmentation on the genetic structure of *Polyommatus coridon *(Lepidoptera: Lycaenidae): implications for conservation. Biol Conserv.

[B27] Slate J, Kruuk LEB, Marshall TC, Pemberton JM, Clutton-Brock TH (2000). Inbreeding depression influences lifetime breeding success in a wild population of red deer (*Cervus elaphus*). Proc R Soc Lond B Bio.

[B28] Charpentier M, Setchell JM, Prugnolle F, Knapp LA, Wickings EJ, Peignot P, Hossaert-McKey M (2005). Genetic diversity and reproductive success in mandrills (*Mandrillus sphinx*). Proc Natl Acad Sci USA.

[B29] Ortego J, Aparicio JM, Cordero PJ, Calabuig G (2008). Individual genetic diversity correlates with the size and spatial isolation of natal colonies in a bird metapopulation. Proc R Soc Lond B.

[B30] Krauss J, Schmitt T, Seitz A, Steffan-Dewenter I, Tscharntke T (2004). Effects of habitat fragmentation on the genetic structure of the monophagous butterfly *Polyommatus coridon *along its northern range margin. Mol Ecol.

[B31] Binzenhofer B, Biedermann R, Settele J, Schroder B (2008). Connectivity compensates for low habitat quality and small patch size in the butterfly *Cupido minimus*. Ecol Res.

[B32] Cassel-Lundhagen A, Sjogren-Gulve P (2007). Limited dispersal by the rare scarce heath butterfly – potential consequences for population persistence. J Insect Conserv.

[B33] Fischer J, Lindenmayer DB (2007). Landscape modification and habitat fragmentation: a synthesis. Global Ecol Biogeogr.

[B34] Bender DJ, Contreras TA, Fahrig L (1998). Habitat loss and population decline: A meta-analysis of the patch size effect. Ecology.

[B35] Schtickzelle N, Baguette M (2003). Behavioural responses to habitat patch boundaries restrict dispersal and generate emigration-patch area relationships in fragmented landscapes. J Anim Ecol.

[B36] Schtickzelle N, Mennechez G, Baguette M (2006). Dispersal depression with habitat fragmentation in the bog fritillary butterfly. Ecology.

[B37] Baguette M, Van Dyck H (2007). Landscape connectivity and animal behavior: functional grain as a key determinant for dispersal. Land Ecol.

[B38] Schtickzelle N, Joiris A, Van Dyck H, Baguette M (2007). Quantitative analysis of changes in movement behaviour within and outside habitat in a specialist butterfly. BMC Evol Biol.

[B39] Burnham KP, Anderson DR (2002). Model Selection and Multimodel Inference: A Practical Information-Theoretic Approach.

[B40] White GC, Burnham KP (1999). Program MARK: survival estimation from populations of marked animals. Bird Study.

[B41] Schtickzelle N, Le Boulengé E, Baguette M (2002). Metapopulation dynamics of the bog fritillary butterfly: demographic processes in a patchy population. Oikos.

[B42] Zietkiewicz E, Rafalski A, Labuda D (1994). Genome fingerprinting by Simple Sequence Repeat (Ssr)-anchored polymerase chain-reaction amplification. Genomics.

[B43] Vandewoestijne S, Baguette M (2002). The genetic structure of endangered populations in the Cranberry Fritillary butterfly, *Boloria aquilonaris *(Lepidoptera, Nymphalidae): RAPDs *vs *allozymes. Heredity.

[B44] Lynch M, Milligan BG (1994). Analysis of population genetic-structure with RAPD markers. Mol Ecol.

[B45] Schneider S, Roessli D, Excoffier L (2000). Arlequin ver 2000: A software for population genetic data analysis.

[B46] Moilanen A, Nieminen M (2002). Simple connectivity measures in spatial ecology. Ecology.

[B47] Butaye J, Honnay O, Adriaens D, Delescaille LM, Hermy M (2005). Phytosociology and phytogeography of the calcareous grasslands on Devonian limestone in Southwest Belgium. Belg J Bot.

[B48] Ellenberg H Zeigerwerte der Gefässpflanzen Mitteleuropas Scripta Geobotanica IX.

[B49] Roff DA (2006). Introduction to Computer-intensive Methods of Data Analysis in Biology.

